# Shiga Toxin, Stx2e, Influences the Activity of Porcine Lymphocytes In Vitro

**DOI:** 10.3390/ijms24098009

**Published:** 2023-04-28

**Authors:** Daniel Sperling, Hana Stepanova, Han Smits, Anne-Kathrin Diesing, Martin Faldyna

**Affiliations:** 1 Ceva Sante Animale, 33500 Libourne, France; 2 Veterinary Research Institute Brno, 621 00 Brno, Czech Republic; 3 SID—Science and Investigation Department, 33500 Libourne, France; 4 Ceva Innovation Center GmbH, 06861 Dessau-Rosslau, Germany

**Keywords:** swine, oedema disease, *E. coli* (STEC), immunosuppression, lymphocyte activity

## Abstract

Oedema disease (OD) in piglets is one of the most important pathologies, as it causes significant losses due to the high mortality because of the Shiga toxin family, which produces *Escherichia coli* (STEC) strains. The main toxin responsible for the characteristic pathologies in pigs is Shiga toxin 2 subtype e (Stx2e). Moreover, there is growing evidence that Stx’s family of toxins also targets immune cells. Therefore, this study evaluated the effect of different concentrations of Stx2e on porcine immune cells. Porcine peripheral blood mononuclear cells were pre-incubated with Stx2e, at three different concentrations (final concentrations of 10, 500, and 5000 CD50/mL) and with a negative control group. Cells were then stimulated with polyclonal mitogens: concanavalin A, phytohemagglutinin, pokeweed mitogen, or lipopolysaccharides. Cell proliferation was assessed by BrdU (or EdU) incorporation into newly created DNA. The activation of the lymphocyte subsets was assessed by the detection of CD25, using flow cytometry. The toxin significantly decreased mitogen-driven proliferation activity, and the effect was partially dose-dependent, with a significant impact on both T and B populations. The percentage of CD25+ cells was slightly lower in the presence of Stx2e in all the defined T cell subpopulations (CD4+, CD8+, and γδTCR+)—in a dose-dependent manner. B cells seemed to be the most affected populations. The negative effects of different concentrations of Stx2e on the immune cells in this study may explain the negative impact of the subclinical course of OD.

## 1. Introduction

The Shiga toxin family, which is produced by Shiga toxin-producing *E. coli* (STEC) strains, only causes clinical diseases in a limited number of species: haemorrhagic colitis or the haemolytic-uremic syndrome (HUS) in humans, and oedema disease (OD) in nursery piglets. In piglets, OD is one of the most important pathologies, as it causes significant losses due to its high mortality rate. It also has a negative effect on production results and the cost of the associated antibiotic treatments is high [1]. The clinical disease is characterised by neurologic symptoms, sudden death, and gross oedema in predilection sites and organs: the eyelids, intestinal tract, mesentery and the microscopic arteriolar necrosis [2,3]. Pigs with subclinical disease often have necrosis in the arterioles of the intestinal tract and brain and a lower weight, which negatively affects the productive performance of the affected piglets [4,5].

The main toxin responsible for pathologies in piglets is Shiga toxin 2 subtype e (Stx2e) [6,7]. The structure of Stx2e has been described in previous studies: it consists of one A subunit and five B subunits [8]. The A subunit has N-glycosidase activity, which specifically cleaves to an adenosine base in the 28S rRNA of the 60 S ribosomal subunits. This inhibits protein synthesis in the target cells. The B subunits bind to the glycolipid receptor, or to the globotriaosylceramide (Gb3) or the globotetraosylceramide (Gb4) receptors on the target cells where, through endocytosis, the toxin is internalized into the cells [9]. The receptors were detected on many cell types, including the peripheral blood leukocytes, especially on monocytes, granulocytes, and alveolar macrophages [10,11].

The Stx2e-mediated destruction and necrosis of endothelial cells in venules and arterioles result in thrombotic microangiopathy, the histological hallmark of diseases [4,12]. However, there is growing evidence that Stx’s family of toxins also targets the immune cells of the host [13]. The human B cells of the immunoglobulins G- and A-committed subsets have been reported as being highly susceptible to the cytotoxic action of Shiga toxin 1 (Stx1) [14]. There are previous reports that have confirmed that Stx1 binds to isolated human and bovine leukocytes and that it suppresses the activation and proliferation of lymphocytes [11,13,15,16,17]. Therefore, a similar negative effect could be expected in the case of porcine immune cells, as Stx2e was detected in the red cell fraction of the blood from some of the pigs with clinical OD and in some that were also asymptomatic.

The previously conducted study provided direct evidence that Stx can bind to porcine leukocytes [11]. In the study, Stx bound to PBL (monocytes and granulocytes), to tissue leukocytes (alveolar macrophages), and to tissue locations where leukocytes reside (i.e., the intestinal lamina propria, intestinal lymphoid aggregates, and the liver).

Importantly, the sample of whole blood exhibited the strongest interaction with Stx2e, which was in agreement with the high content of Gb4Cer [10]. This observation might have a functional impact on the putative role of blood cells as possible transport vehicles of Stx2e through the circulation of infected animals; however, the direct negative effect of the toxin on immune cells cannot be excluded.

There is also a subclinical form of OD, which was described under the challenge trial that was set up, and also under the field conditions of commercial farms [4,5]. The potential negative effects of sublethal concentrations of Stx2e on immune cells may explain the negative effects observed under subclinical conditions, under the field conditions. This may also explain the negative effects on the production results, which are associated with elevated mortality rates and an increased need for antimicrobial treatment, due to the associated secondary infections. Therefore, the objective of our study was to assess the effect of Stx2e on porcine immune cells.

## 2. Results

### 2.1. Proliferation Activity

**Figure 1:**
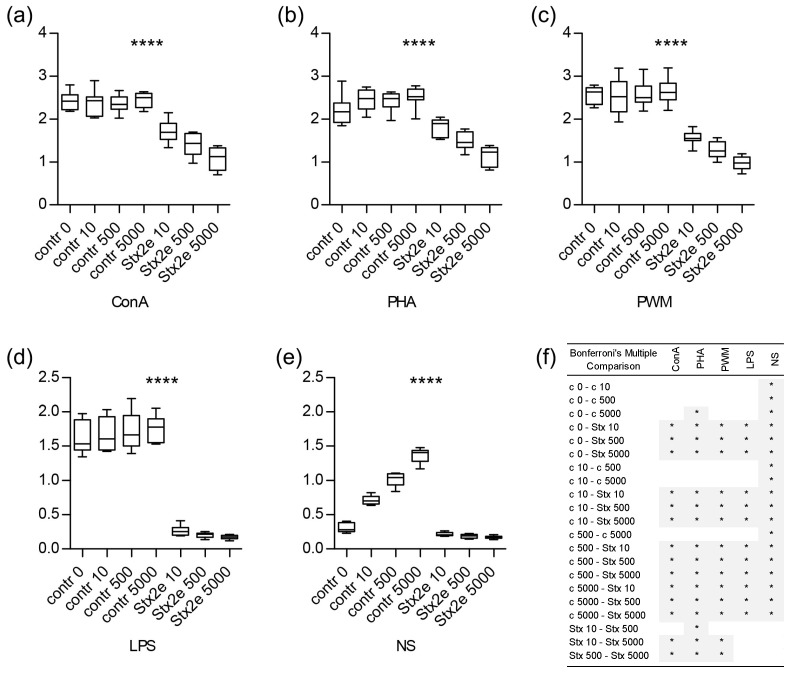
Proliferation activity. Evaluated by BrdU incorporation after stimulated with polyclonal mitogens including concanavalin A (ConA; (**a**)), phytohemagglutinin (PHA; (**b**)), pokeweed mitogen (PWM, (**c**)) and lipopolysaccharides (LPS; (**d**)) and non-stimulated cells (NS; (**e**)). Previously, cells were pre-incubated with Stx2e at three different concentrations: 10 (Stx2e 10), 500 (Stx2e 500), and 5000 (Stx2e 5000) CD50/mL or with negative control (mock) at the same dilution (contr 10, contr 500, contr 5000). Cells with medium only were left as a negative control sample (contr 0). Boxplots represent the median (line), first and third quartiles (box), and minimum and maximum (whiskers) of absorbance values, n = 8. Data were analyzed using repeated measures ANOVA, statistical significance is marked in graphs as *p*-values summary (**** *p* ≤ 0.0001). Multiple comparison of all pairs was evaluated by Bonferroni’s Test and is summarized in the table (**f**) (white, *p* > 0.05; grey, * *p* ≤ 0.05).

Cell proliferation was assessed by BrdU incorporation into newly created DNA. Four different mitogens (ConA, PHA, PWM, and LPS) were used and non-stimulated cells (NS) were also included (Figure 1). The toxin significantly decreased proliferation activity in all cultures (ConA, PHA, PWM, and LPS: *p*-value < 0.0001, repeated measures ANOVA, Figure 1). Bonferroni’s multiple comparison tests were used to compare all of the pairs, summarized in Figure 1. The effects of Stx2e were partially dose-dependent. Samples that were incubated with the negative control (mock) at three dilutions (alternative to the Stx2e dilution) did not differ from the cells that were incubated with medium only (“ctrl 0”), in which the samples were stimulated with ConA, PHA, PWM, or LPS. In contrast, we found higher proliferation activity in the presence of the mock, in which a significant dose-dependent trend was found. A possible explanation for this is the possible presence of LPS in the mock originating from *E. coli* culture used for recombinant Stx2e preparation. Because the negative control background was not significant in the mitogen-stimulated samples, it could be considered minor in the evaluation of the effects of the Stx2e toxin. Based on the data obtained, the highest dilution (“ctrl 5000”) of the mock was selected as the control without Stx2e for the other methods included in the study.

### 2.2. Viability of Cells after Stx2e Exposure

**Figure 2:**
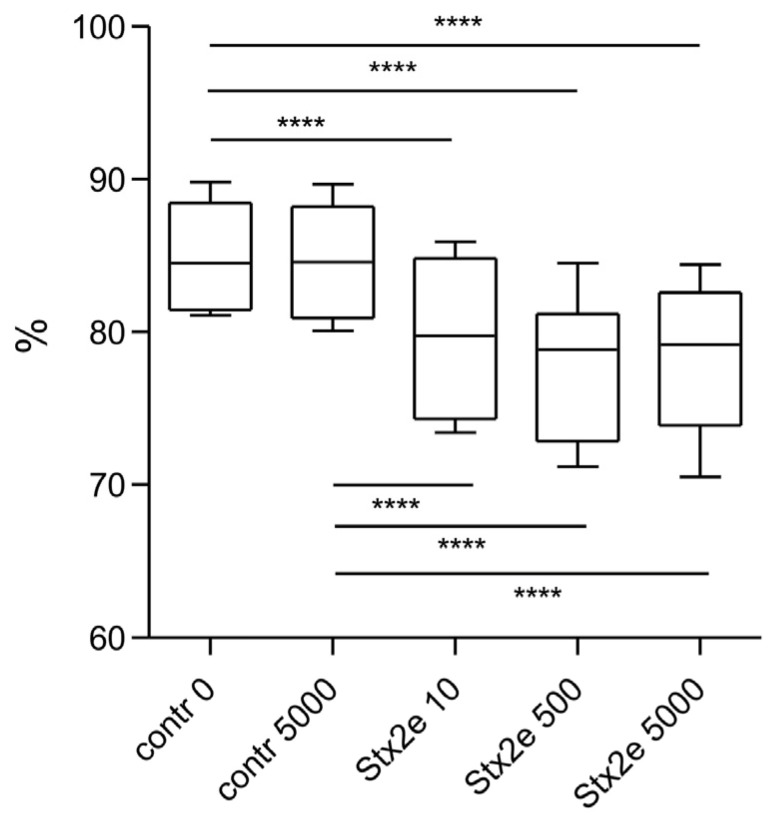
Viability of Cells after Stx2e Exposure. Evaluated by flow cytometry after propidium iodide (PI) staining. PBMC without mitogens were exposed to Stx2e at three different concentrations: 10, 500, and 5000 CD50/mL or with mock at the highest concentration (contr 5000) and medium only (contr 0) as negative controls. Percentage of live cells (PI negative) is presented by boxplots: median (line), first and third quartiles (box), and minimum and maximum (whiskers), n = 8. Repeated measures ANOVA with Bonferroni’s Multiple Comparison Test were considered to indicate significant differences (**** *p* ≤ 0.0001).

The viability of PBMC after cultivation was evaluated using propidium iodide (PI), which selectively stains cells with a permeabilized membrane. The percentage of live cells (PI negative) was analyzed according to the gating strategy (supplementary data, Figure S1). The toxin had a significant impact on the cells’ viability in all types of cultures (*p* value < 0.0001, repeated measures ANOVA samples). The samples cultured with Stx2e showed a significantly lower percentage of live cells, with a minor dose-dependent relationship (Figure 2). There was no significant difference between the control that was cultured with medium only and the mock as negative control. The mitogen-stimulated samples showed the same result of viability as the unstimulated samples.

### 2.3. CD25 Expression on Lymphocyte Subpopulations

The activation of particular lymphocyte subsets was assessed through the detection of early markers of lymphocyte activation, CD25, using multicolor flow cytometry. B lymphocytes (CD3-sIgM+) and three subpopulations of T lymphocytes (CD3+) were defined as CD3+ CD4+ CD8+/− (CD4+); CD3+ CD4− CD8+ (CD8+); and CD3+ γδTCR+ (γδTCR+). The expression of CD25 in these particular populations was analyzed. These populations were gated from live cells, according to the gating strategy shown in supplementary data (Figure S2).

**Figure 3:**
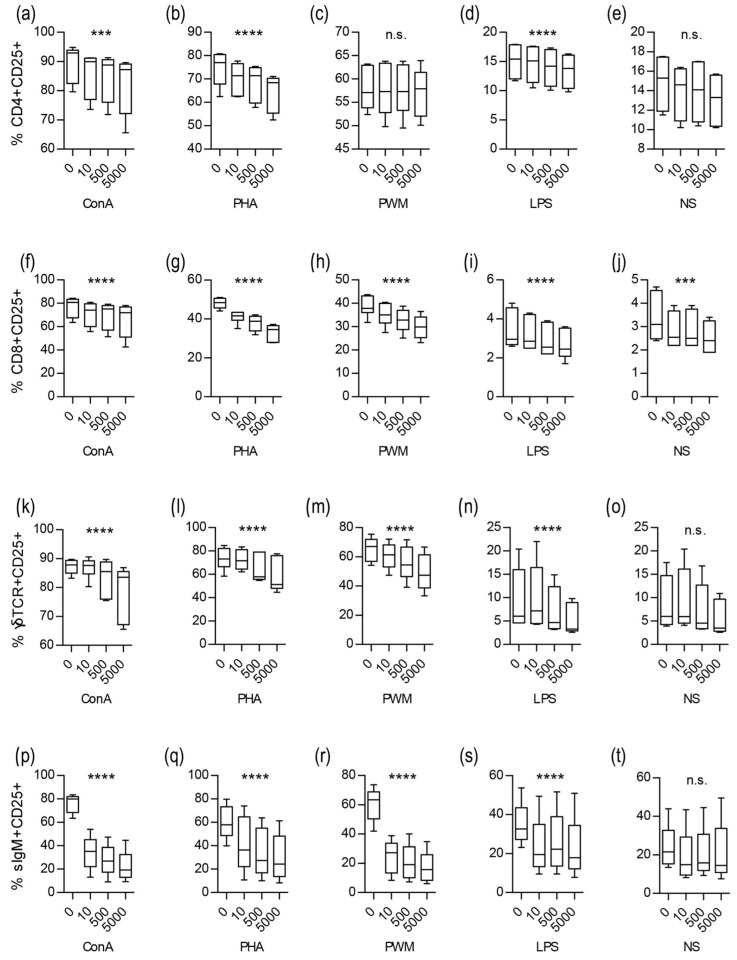
CD25 Expression on Lymphocyte Subpopulations. Four subpopulations of T lymphocytes and B lymphocytes were defined as CD3+ CD4+ CD8+/− (CD4+; (**a**–**e**)), CD3+ CD4− CD8+ (CD8+; (**f**–**j**)), CD3+ γδTCR+ (γδTCR+; (**k**–**o**)), and CD3-sIgM+ (sIgM+; (**p**–**t**)). The x-axis represents Stx2e concentration 0, 10, 500, and 5000 CD50/mL. Cells were pre-incubated (2 h) with Stx2e and subsequently stimulated with polyclonal mitogens including concanavalin A (ConA; (**a**,**f**,**k**,**p**)), phytohemagglutinin (PHA; (**b**,**g**,**l**,**q**)), pokeweed mitogen (PWM; (**c**,**h**,**m**,**r**)), and lipopolysaccharides (LPS; (**d**,**i**,**n**,**s**)). Cells without mitogen were left as a non-stimulated samples (NS; (**e**,**j**,**o**,**t**)). Boxplots represent percentage of CD25+ cells from particular populations: median (line), first and third quartiles (box), and minimum and maximum (whiskers), n = 6. Data were analyzed using repeated measures ANOVA, statistical significance is marked in graphs as *p*-values summary (n.s. *p* > 0.05; *** *p* ≤ 0.001; **** *p* ≤ 0.0001).

The toxin had a significant impact on both T and B populations. The percentages of CD25+ cells were slightly lower in the presence of Stx2e in all of the defined T cell subpopulations (CD4+, CD8+, and γδTCR+), with a dose-dependent relationship. Statistically significant differences were found for nearly all of the cultures and are summarised in Figure 3. The B cells defined as CD3-sIgM+ were the populations that were most affected by Stx2e. Within B lymphocytes, CD25 expression induced by mitogen stimulation was almost completely inhibited by toxin treatment. The toxin-induced a significant decrease in the percentage of IgM+ CD25+ cells in the cultures that were stimulated with all the mitogens used in the study (Figure 3).

### 2.4. Proliferation Activity of Particular Lymphocyte Subsets

**Figure 4:**
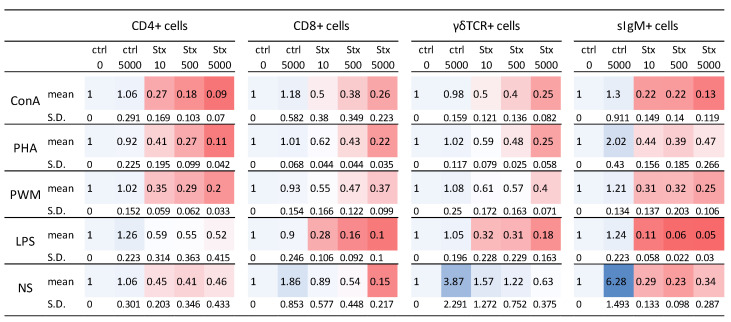
Proliferation of Particular Lymphocyte Subsets. Incorporation of 5-Ethynyl-2′-deoxyuridine (EdU) into newly created DNA measured by flow cytometry. Cells were pre-incubated (2 h) with Stx2e and subsequently stimulated with polyclonal mitogens including concanavalin A (ConA), phytohemagglutinin (PHA), pokeweed mitogen (PWM) and lipopolysaccharides (LPS). Cells without mitogen were left as a non-stimulated sample (NS). Four subpopulations of T lymphocytes and B lymphocytes were defined as CD3+ CD4+ CD8+/− (CD4+), CD3+ CD4− CD8+ (CD8+), CD3+ γδTCR+ (γδTCR+), and CD3-sIgM+ (sIgM+). Percentage of EdU-positive cells was evaluated for particular subpopulations. For data presentation, the ratio was calculated as: “control cells with medium only (contr 0)”/“cells cultured with Stx2e (or mock as ctrl 5000)”. The data are presented as a heat map in red (ratio < 1) or blue color (ratio > 1). Data are presented as mean and S.D. (n = 3).

The proliferation of particular lymphocyte subsets was assessed by the incorporation of 5-Ethynyl-2′-deoxyuridine (EdU) into newly created DNA and subsequently measured by flow cytometry. The three subpopulations of T lymphocytes (CD3+) and B lymphocytes (CD3−) were defined as CD3+ CD4+ CD8+/− (CD4+); CD3+ CD4− CD8+ (CD8+); CD3+ γδTCR+ (γδTCR+); and CD3-sIgM+ (sIgM+) according to gating strategy (Supplementary Data, Figure S2). The percentage of EdU-positive cells was evaluated for particular subpopulations. Increased proliferation activity was detected within samples incubated with mock at the highest concentration (ctrl 5000). This observation is in accordance with the proliferation data presented in Figure 1. A possible explanation is the presence of LPS in the mock originating from the *E. coli* culture used for recombinant Stx2e preparation. As summarized in Figure 4, the method confirmed that Stx2e negatively affected the proliferation activity of cells after non-specific stimulation with different mitogens (ConA, PHA, PWM, or LPS). This is in agreement with data obtained by the BrdU-based method. The added value of the EdU-based method is the distinction of individual populations. A decreased proliferation activity was confirmed for all of the subsets included in the study with the strongest impact on CD4+ T cells and IgM+ B cells.

## 3. Discussion

Some pathogens have developed escape mechanisms that help them to prolong their persistence in a host organism. Among these escape mechanisms are active modulation and interfering with the immune system. These are the most important and frequently used mechanisms. Respective bacterial strains have acquired and developed mechanisms to modulate the immune response of their hosts [18,19]. The heat-labile enterotoxins (LT) of *Escherichia coli* and the cholera toxin (CT) of *Vibrio cholerae*, from the group of toxins possessing a 5B-plus-A basic structure, are among the best characterized bacterial immunomodulators [18]. The ability of STEC strains to cause persistent infections was confirmed in gnotobiotic piglets experimentally and in conventional piglets under field conditions, where animals from positive farms can be colonized and affected for periods of up to several weeks after the initial intestinal infection [20,21]. Stx toxin family-induced immunomodulation has been proposed by several authors [18,20,22,23].

The aim of our study was to test whether Stx2e, as a major factor in the virulence of STEC, can influence different subpopulations of lymphocytes, isolated from the peripheral blood of nursery pigs, in vitro. Their ability to become activated was assessed by mitogen-driven proliferation and by the detection of early markers of lymphocyte activation (CD25) using multicolor flow cytometry. The molecule CD25 represents an activation molecule that marks functionally distinct subsets and was a suitable target for the analysis of the toxin’s effect. It was successfully used in previous studies [24]. The three subpopulations of T lymphocytes (CD3+) and B lymphocytes (CD3-) in this study were defined as CD3+ CD4+ CD8+/− (helper T lymphocytes); CD3+ CD4− CD8+ (cytotoxic T lymphocytes); CD3+ γδTCR+ (γδTCR T lymphocytes); and CD3-sIgM+ (B lymphocytes) to represent the relevant cell components of the adaptive immune response system in pigs [25]. The lymphocyte population has a different responsibility to the mitogens, based on their different modes of stimulation; consequently, a complete panel (ConA, PHA, PWM, and LPS) was used in the study.

In this study, the effects of three different toxin concentrations on the immune cells and their subpopulations were investigated. The selected concentrations of the Stx2e toxin corresponded to the Stx2 toxin secretion of the clinical STEC strains that were isolated from the nursery pigs on farms, both with and without a history of OD [26]. Based on their ability and the amount of toxin produced, those strains were identified as low, moderate, and high secretors, with concentrations ranging from 62–7152 CD50/mL [26]. In this respect, the concentrations selected in our study corresponded to the concentrations in the fields.

Our results suggest that Stx2e has a negative effect on porcine immune cells, due to the toxin’s ability to compromise the proliferating activity of lymphocytes; moreover, the effect of Stx2e was partially dose dependent. The reduced functional activity of the lymphocytes, after the mitogen activation, from the piglets infected with the STEC strain has previously been described [20]. The difference between the control piglets (inoculated by STEC—negative *E. coli*) and the infected group was significant, which highlights the negative effects produced by the Shiga toxin in STEC-colonised piglets [20]. This effect was seen to compromise the immune responsive capacity of its host under in vivo conditions. A similar effect on different lymphocyte subsets (PMBC) in cattle with different sensitivity to Stx1 in vitro has also been described in other studies [13,18,27].

In our study, the porcine B cells, defined as sIgM+, seemed to be the populations that were most affected by Stx2e. The toxin-induced a significant decrease in the percentage of IgM+ CD25+ cells in the cultures stimulated with all the mitogens used in this study. In other studies, a similar effect was also observed in humans, where Stxs only appeared to target the B cell compartment [14,28]. A possible targeting of B cells was also observed in another study, in which a decreased antibody response and a lower production of complement-fixing antibodies were observed in pigs that had been inoculated by the STEC strain [20].

The toxin had a significant negative impact on cell viability in our study, which showed a significantly lower percentage of live cells with a minor dose-dependent relationship. This evidence for such a negative effect is supported by observations in previous studies, in which both histologic changes in lymphoid tissues (atrophy of lymphoid tissue) and in the total lymphocyte count (decreased numbers of peripheral lymphocytes) was observed in piglets infected with the STEC strain [20]. In addition to the primary pathogenic mechanism of Stx-mediated damage that has been proposed—i.e., direct Stx toxin cytotoxicity for vascular endothelial on predilection sites—the specific cytotoxicity targeting immune cells may also play a role in immunosuppression [18]. The indirect pathogenic mechanisms of an STEC infection, based on Stx-induced immunomodulation, have been proposed by several other authors, who have conducted both in vitro and in vivo studies in species other than swine, including humans [14,20,23,29,30].

Gb3 is expressed in many cell types, including tubular endothelium and other kidney cells [31,32]. This expression is responsible for the clinical picture of HUS. Other cell populations expressing Gb3 include the nervous system [33]. Gb3 has also been observed on many hematopoietic cells. The expression of Gb3 on lymphocytes was observed to be higher on the lymphocytes from the tonsils than from the peripheral blood or thymus [34]. Only 4% of the expression that is attributed to blood leucocytes belongs to lymphocytes [35]. Among the lymphocytes, the highest expression is on the B lymphocytes.

Specifically, in pigs, novel porcine Stx (both Stx1 and Stx2) binding sites have previously been identified, including kidney tubules, intestinal lymphoid aggregates, sinusoidal cells in the liver, and isolated leukocytes [11]. When Stx is bound to porcine PBL (monocytes and granulocytes), tissue leukocytes (alveolar macrophages), and to tissue locations where leukocytes reside, these tissues might be involved in Stx-induced immunomodulation, transport, or in the clearance of Stx [11].

One of the potential roles of Gb3 expression is its involvement in the FAS receptor–ligand system [36]. This is one of the mechanisms by which infected cells are destroyed by NK cells or cytotoxic T lymphocytes [37].

From a functional point of view, Gb3 serves as a receptor for bacteria or their toxins [38,39,40]. This attachment is associated with lipid–raft-mediated endocytosis, which triggers many intracellular signals [41]. They have been summarized in a review by Lingwood [42]. One of the consequences of these downstream pathways is the ability of a subunit to block protein synthesis using its RNA glyconase activity [43]. It may be beyond the ability of Stx2e to suppress immune responses and to establish persistent infections in sensitive piglets.

Collectively, our results may explain the possible negative effects of sublethal doses of the Stx toxin on immune effector cells, which might be one of the pathological mechanisms involved in subclinical OD, described both experimentally and also at the field level [4,5]. The benefit of vaccination against the Stx2 toxin, using the recombinant toxoid vaccine, is a reduction in classical cases of OD and its consequent mortality rate [1]. In addition, this vaccination can also reduce the number of cases involving clinical signs and pathologies that are not directly connected with, or typical to, OD, for example, respiratory problems and lameness [44]. Our study has shown that the production of Stx toxin-neutralizing antibodies can be beneficial, even in subclinical cases, by blocking the immunocompromising effects of the toxin.

## 4. Material and Methods

### 4.1. Stx2e and Control Preparation

The recombinant Stx2e toxin applied in this study was derived from a plasmid containing the wild-type Stx2e A and B genes, expressed by an *E. coli* K12 strain. The culture was grown in LB medium (Difco™ LB Broth Lennox by Becton, Dickinson, and Company, Franklin Lakes, NJ, USA, 5 g/L yeast extract, 10 g/L tryptone (casein digested by trypsin), 5 g/L NaCl). It was broken by ultrasonication, centrifuged, and the supernatant was sterile filtered. The recombinant Stx2e toxin was diluted with sterile PBS to a final concentration of 1 Mio (10^6^) CD50/mL and stored at −80 °C. Prior to use, the thawed rStx2e material was diluted with physiological saline (NaCl), to the final concentrations of 10, 500, and 5000 CD50/mL.

The negative control applied derived from an *E. coli* K12 strain without any plasmid, the strain carries no Stx2e genes. The culture was performed the same way as described for Stx2e toxin preparation. It was centrifuged and the supernatant was sterile filtered. Prior to use, the thawed culture supernatant was diluted with physiological saline (NaCl), to the equivalences of the final concentrations of the preparations of Stx2e with 10, 500, and 5000 CD50/mL.

### 4.2. Animals, Cell Isolation, and Culture

Animals (end nursery age) used in the study were purchased from a farm without a history of edema disease and were subjected to repeated bacteriological examinations for *E. coli* isolation and strain typisation—detection of virulence factors including Stx2e and F18 adhesin, with negative results. Animal care and use protocols were approved by the Ethical Committee of the Veterinary Research Institute, according to guidelines set out in the Animal Protection Act and were subsequently approved by the Branch Commission for Animal Welfare of the Czech Republic’s Ministry of Agriculture (reference number 31778/2020-MZE-18134). Blood was collected from the jugular vein of six clinically healthy pigs. Blood samples were heparinized using 25 IU/mL of sodium heparin (Zentiva, Prague, Czech Republic). Peripheral blood mononuclear cells (PBMCs) were isolated by gradient centrifugation using Histopaque-1077 (Sigma-Aldrich, St. Louis, MO, USA). Purified PBMC were cultured in 96-well plates, at a concentration of 5 × 10^5^ cells per well, in 1 mL of RPMI, 1640 medium with antibiotics (100 IU/mL penicillin and 100 μg/mL streptomycin) and supplemented with 10% fetal bovine serum (FBS). Cells were pre-incubated for 2 h with Stx2e—provided by Ceva Innovation Centre, Dessau, Germany—at three different concentrations: 10, 500, and 5000 CD50/mL, or with mock as negative control (*E. coli* K12 strain broken by ultrasonication, centrifuged and the supernatant sterile filtered) at the same dilution as Stx2e or with medium only. Afterward, cells were stimulated with polyclonal mitogens, including concanavalin A (ConA: 5 µg/mL), phytohemagglutinin (PHA: 40 µg/mL), pokeweed mitogen (PWM: 10 µg/mL), and lipopolysaccharides (LPS: 25 µg/mL). PBMC without mitogens were left as non-stimulated samples (NS). The cells were incubated at 37 °C in 5% CO_2_, according to the methods described below.

### 4.3. Proliferation Activity Assay

Samples of isolated PBMC were incubated overnight in duplicates with Stx2e toxin and mitogens. Next, bromodeoxyuridine (BrdU) was added (10 µM) for the next 18–20 h. Plates were centrifuged after incubation and the medium was removed. The plates were subsequently dried off (60 min, 60 °C) and stained, according to the manufacturer’s recommendations (Cell Proliferation ELISA BrdU—colorimetric, Roche, Mannheim, Germany). The absorbance of each individual well was measured at 450 nm, using an ELISA reader (Infinite M.nano Tecan, Biotec Instruments Inc., Agilent Technologies, Santa Clara, CA, USA). The results were presented as the mean of absorbance from duplicates.

### 4.4. Flow Cytometry Analysis of Viability and CD25 Expression in Lymphocyte Subpopulations

The mitogen-driven activation of particular lymphocyte populations was performed after overnight PBMC cultivation, in different Stx2e concentrations. The expressions of surface markers CD3, CD4, CD8, γδTCR, sIgM, and CD25 were measured by flow cytometry. The primary mouse anti-pig antibodies were as follows: CD3ε (PerCP-Cy™5.5-conjugated, BB23-8E6-8C8, BD Biosciences, Franklin Lakes, NY, USA); γδTCR (unconjugated, PGBL22A, IgG1, WSU, Pullman, WA, USA); CD4 (unconjugated, 10.2H2, IgG2b, WSU); CD8α (unconjugated, 76-2-11, IgG2a, WSU); CD25 (unconjugated, PGBL25A, IgG1, WSU); and sIgM (unconjugated, PG145A, WSU). As secondary antibodies, isotype-specific, fluorochrome-labeled goat anti-mouse antibodies were used: Alexa Fluor 488; Alexa Fluor 647 Invitrogen (Thermo Fisher Scientific, Invitrogen, Waltham, MA, USA); and Brilliant Violet 421 (Jackson Immuno Research, Cambridgeshire, UK). Flow cytometry was performed using an LSR Fortessa flow cytometer, operated by Diva software, version 6.0 (Becton Dickinson, Franklin Lakes, NJ, USA). Doublets (defined by plotting the width against the area of forward scatter) and dead cells (stained with propidium iodide, PI) were excluded from the CD25 analysis. Viability was evaluated from the single-cell gate, as PI negative population according to the gating strategy presented in supplementary data (Figure S1, Gating strategy–viability). The percentage of cells positive for the CD25 marker was evaluated for particular populations, defined as follows: CD3+ CD4+ CD8+/− (CD4+), CD3+ CD4− CD8+ (CD8+), CD3+ γδTCR+ (γδTCR+), and CD3-sIgM+ (sIgM+). The gating strategy is shown in supplementary data (Figure S2, Gating strategy–T and B lymphocyte subpopulations).

### 4.5. Flow Cytometry Analysis of Proliferation Activity in Lymphocyte Subpopulations

The mitogen-driven proliferation of particular lymphocyte populations was performed by flow cytometry. Samples were incubated with Stx2e toxin and mitogens overnight. Next, 5-Ethynyl-2′-deoxyuridine (EdU) was added (10 µM) for the next 18–20 h. Cells were harvested and stained for surface markers, including CD3, CD4, γδTCR, and sIgM, as described above. Subsequently, samples were tested by Click-iT EdU cell proliferation assays (Thermo Fisher Scientific, Invitrogen), according to the manufacturer’s recommendations. Flow cytometry was performed using an LSR Fortessa flow cytometer, operated by Diva software, version 6.0 (Becton Dickinson). The percentage of EdU-positive cells was evaluated for particular subpopulations, defined as CD3+ CD4+ CD8+/−; CD3+ CD4− CD8+; CD3+ γδTCR+; and CD3-sIgM+ (hereinafter referred to as CD4+; CD8+; γδTCR+; and sIgM+, respectively). For the presentation of the data, the ratio was calculated as “control cells with medium only (ctrl 0)”/“cells cultured with Stx2e (or mock)”.

### 4.6. Statistical Analysis and Data Presentation

Data were analyzed using repeated measures ANOVA, with Bonferroni’s Multiple Comparison Test—*p*-values < 0.05 were considered to indicate significant differences. Outliers were excluded from the analysis, according to the Grubbs test. Statistical significance was presented as *p*-values and/or *p*-values summary (n.s. *p* > 0.05; * *p* ≤ 0.05; ** *p* ≤ 0.01; *** *p* ≤ 0.001; **** *p* ≤ 0.0001). Statistical analysis was performed, and graphs were created using GraphPad Prism software (GraphPad Software, version 5.04, San Diego, CA, USA). Samples were marked as follows: cells cultured in medium only, without Stx2e—“ctrl 0”; cells with mock (*E. coli* K12 strain broken by ultrasonication, centrifuged, and the supernatant sterile filtered) at three dilution alternatives to Stx2e concentration—hereinafter referred to as “ctrl 10”, or “ctrl 500”, or “ctrl 5000”; and cells with Stx2e at concentrations 10, 500, and 5000 CD50/mL—hereinafter referred to as “Stx2e 10”, or “Stx2e 500”, or “Stx2e 5000”.

## Data Availability

All data are included in this publication.

## Supplementary Materials

The supporting information can be downloaded at: https://www.mdpi.com/article/10.3390/ijms24098009/s1.
