# Supplementary material for: Shiga Toxin, Stx2e, Influences the Activity of Porcine Lymphocytes In Vitro
Source: Int J Mol Sci. 2023 Apr 28;24(9):8009. doi: 10.3390/ijms24098009 (PMC10178452; doi:10.3390/ijms24098009)
Supplement: Supplementary file 1 [file ijms-24-08009-s001.zip › ijms-2345153-supplementary.pdf]

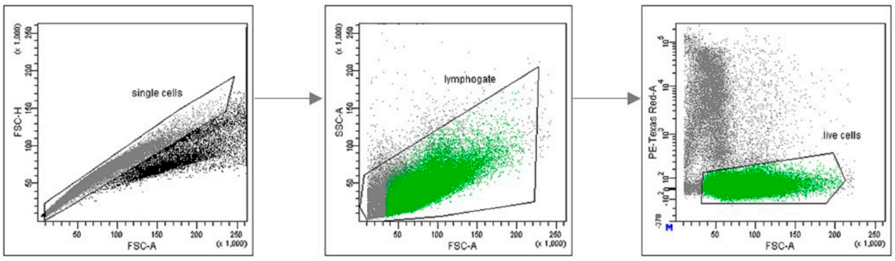

Figure S1. Gating strategy – viability. Single cells were gated according to FSC-A / FSC-H parameters and subsequently propidium iodide negative cells were evaluated as live cells. Representative dotplots from cell culture samples without mitogen are included in the gating strategy.

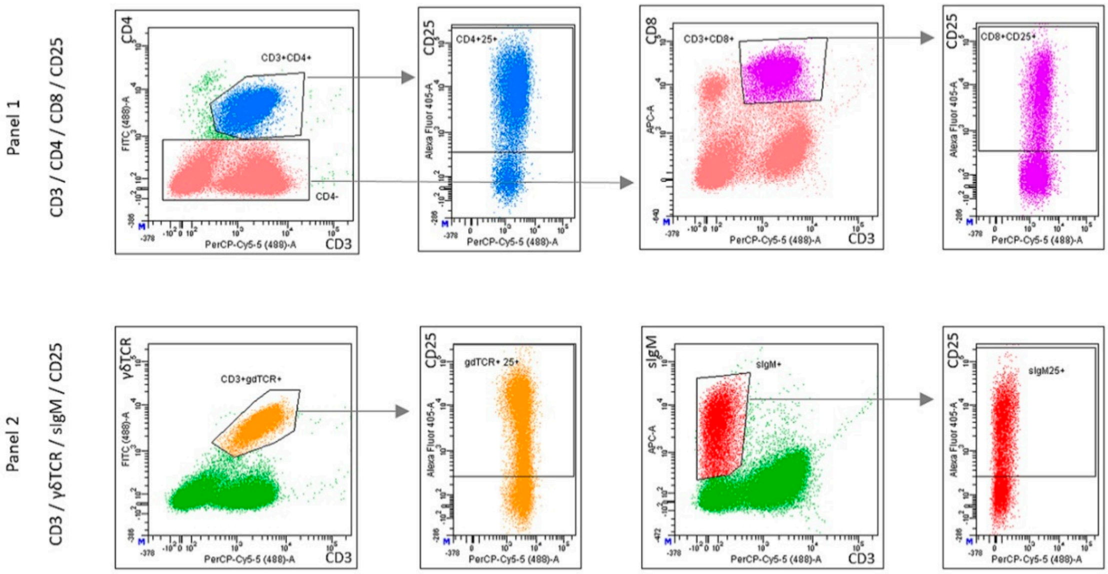

Figure S2. Gating strategy – T and B lymphocyte subpopulations. Surface markers CD3, CD4, CD8, sIgM and CD25 were assessed by flow cytometry. T and B lymphocyte subpopulations were defined from single live cells (Figure S1) and CD25 expression was evaluated. Representative dotplots from cell culture samples without mitogen are included in the gating strategy.
